# Fenofibrate improves vascular endothelial function and contractility in diabetic mice

**DOI:** 10.1016/j.redox.2018.09.024

**Published:** 2018-10-01

**Authors:** Nan Xu, Qin Wang, Shan Jiang, Qijing Wang, Weipeng Hu, Suhan Zhou, Liang Zhao, Lanyu Xie, Jianghua Chen, Anton Wellstein, En Yin Lai

**Affiliations:** a Department of Physiology, School of Basic Medical Sciences, and Kidney Disease Center of First Affiliated Hospital, Zhejiang University School of Medicine, Hangzhou 310058, China; b Department of Reproductive Endocrinology, Women's Hospital, Zhejiang University School of Medicine, Hangzhou 310058, China; c Medical college, Nanchang University, Nanchang 330000, China; d Lombardi Cancer Center, Georgetown University, Washington, DC 20007, USA

**Keywords:** Diabetes, Fenofibrate, Endothelial dysfunction, Nitric oxide, Oxidative stress

## Abstract

Fenofibrate, a peroxisome proliferator-activated receptors α (PPARα) agonist, reduces vascular complications of diabetic patients but its protective mechanisms are not fully understood. Here we tested the hypothesis that fenofibrate improves vascular endothelial dysfunction by balancing endothelium-dependent relaxation and contractility of the aorta in diabetes mellitus (DM). In streptozotocin-induced diabetic mice, eight weeks of fenofibrate treatment (100 mg/Kg/d) improved endothelium dependent relaxation in the macro- and microvessels, increased nitric oxide (NO) levels, reduced renal damage markers and effects of the vasoconstrictor prostaglandin. Levels of superoxide dismutase and catalase were both reduced and hydrogen peroxide was increased in vehicle-treated DM, but these changes were reversed by fenofibrate treatment. Vasodilation of the aorta after fenofibrate treatment was reversed by PPARα or AMPKα inhibitors. Western blots showed that fenofibrate treatment elevated PPARα expression, induced liver kinase B1 (LKB1) translocation from the nucleus to the cytoplasm and activated AMP-activated protein kinase-α (AMPKα), thus activating endothelial NO synthase (eNOS). Also, fenofibrate treatment decreased NF-κB p65 and cyclooxygenase 2 proteins in aortas. Finally, incubation with indomethacin in vitro improved aortic contractility in diabetic mice. Overall, our results show that fenofibrate treatment in diabetic mice normalizes endothelial function by balancing vascular reactivity via increasing NO production and suppressing the vasoconstrictor prostaglandin, suggesting mechanism of action of fenofibrate in mediating diabetic vascular complications.

## Highlights

• Fenofibrate improves diabetic endothelial function is via PPAR/LKB1/AMPK/eNOS signal.
• Fenofibrate reduces diabetic endothelial contractility is via NF-κB/COX-2 pathway.
• Diabetes-associated oxidative stress is attenuated by fenofibrate treatment.

## Graphical abstract

**Figure:**
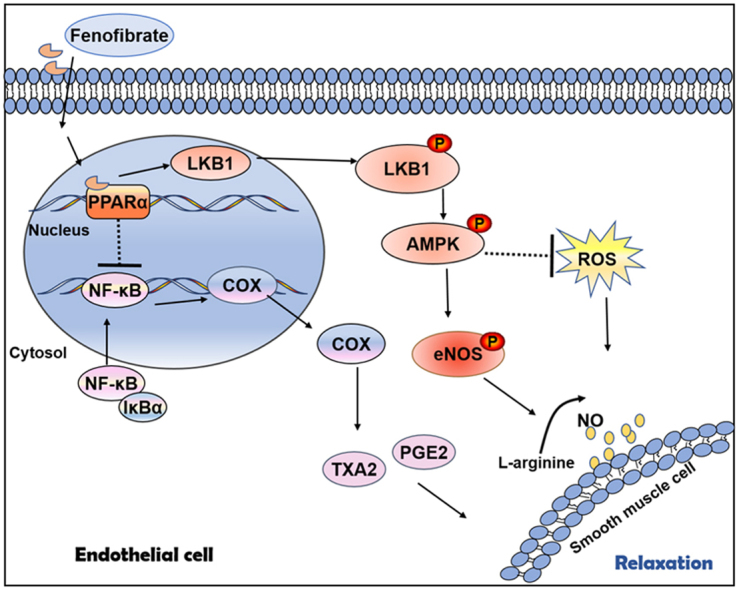

## 1 Introduction

The endothelium and factors derived from endothelial cells are known to control vascular function, including the regulation of vascular tone [1], [2]. Endothelial dysfunction is a hallmark of diabetes and contributes to macrovascular and microvascular complications associated with diabetes [3], [4]. Vascular endothelial dysfunction has been characterized by reduced activity of endothelial nitric oxide synthase (eNOS), decreased generation of nitric oxide (NO) and increased generation of reactive oxygen species (ROS) [3], [5]. In manifest diabetes, there is an imbalance between endothelial-dependent vascular relaxation and constriction, which plays an important role in the development of pathologies associated with the disease [4], [6], [7]. Therefore, understanding the regulation of this imbalance may be important in preventing the development of diabetes-associated vasculopathies.

Fenofibrate is a peroxisome proliferator-activated receptors α (PPAR α) agonist and can improve dyslipidemia [8]. It causes a moderate reduction in total cholesterol (TC), a marked reduction in triglycerides (TG), and an increase in high-density lipoprotein cholesterol (HDL-C) [9]. Emerging clinal evidence has demonstrated that fenofibrate has a therapeutic potential in alleviating diabetes-associated vascular disease independently of its hypolipemic action. The Fenofibrate Intervention and Event Lowering in Diabetes (FIELD) study showed that treatment with fenofibrate (200 mg per day) has promising effects in attenuating the progression of microvascular complications of diabetes, including reduced nonfatal coronary events, decreased need for laser treatment of diabetic retinopathy and delayed progression of diabetic nephropathy [10], [11]. The action to control cardiovascular risk in diabetes (ACCORD) study also demonstrated that fenofibrate, when added to statin therapy, slows the progression of diabetic retinopathy in patients with type 2 diabetes [12]. However, the mechanisms underlying vascular benefits of fenofibrate treatment of patients with diabetes are not fully understood.

Adenosine monophosphate-activated protein kinase (AMPK) has many biological functions, including regulation of glucose metabolism, lipid metabolism [13], intracellular ROS and eNOS levels [14]. Recent studies suggest that modulating AMPK has a great potential in the treatment of metabolic disorders such as obesity and Type 2 diabetes [15]. There are at least two upstream kinases that phosphorylate AMPK: liver kinase B1 (LKB1) and calcium dependent protein kinase kinase-β (CaMKK-β) [16], [17]. Previous studies showed that LKB1 forms a heterotrimeric complex with regulatory proteins, which are required for its activation and cytosolic localization [18]. Experiments in cultured cells demonstrated that fenofibrate plays a role in AMPKα activation [19], [20]. However, data are limited on the effect of fenofibrate on the expression of AMPK and LKB1 in vascular reactivity in diabetes.

Endothelium-dependent contractions are mediated by the products of cyclooxygenase (COX) [21], [22]. COX metabolizes arachidonic acid (AA) to form the unstable prostaglandin H2 which is further converted into thromboxanes (TxAs) and prostaglandins (PGs), including PGE2, PGD2 and prostacyclin [23]. TxA2 can stimulate TxA2/prostanoid (TP) receptor to induce vasoconstriction. TxA2 and PGE2 has been suggested to act as an endothelium derived contracting factor [24]. The production of TxA2 and PGE2 by the endothelium is increased in diabetes [25]. Therefore, inhibition of COX could provide new insights into the mechanism responsible for endothelial dysfunction.

In the present study, we used a mouse model of streptozotocin-induced diabetes mellitus and investigated whether eight-week fenofibrate treatment (100 mg/Kg/d) could prevent diabetes-related endothelial dysfunction in arteries and evaluated possible signaling mechanisms.

## 2 Materials and methods

### 2.1 Animal model and experimental groups

Male adult C57Bl/6 mice (25–28 g) were purchased from SLAC laboratory animal company (Shanghai, China). Animals were treated with standard food and water and housed under climate-controlled conditions with a 12 h light/dark cycle. All the animal handling procedures and experiment protocols were approved by Zhejiang University's institutional animal care and use committee and conducted according to the NIH Guide for the Care and Use of Laboratory Animals.

We induced a diabetes similar to a type I diabetic, according to the protocols of Animal Models of Diabetic Complications Consortium (AMDCC) by the National Institutes of Health. Diabetes was induced by intraperitoneally injection of streptozotocin (STZ, Sigma-Aldrich, St Louis, MO, USA, dissolved in 0.1 M sodium citrate buffer, pH 4.5) at a low-dose (50 mg/kg/day for 5 consecutive days). Hyperglycemia was defined as a random blood glucose level > 16.7 mmol/L three days after last STZ injection. Plasma glucose level was monitored by a contour glucose meter (Roche, Mannheim, Germany) twice a week for eight weeks. Fenofibrate (Sigma, St. Louis, MO, USA), 100 mg/kg/d dissolved in 1% sodium carboxymethyl cellulose, was administered intragastrically to diabetic mice daily for eight weeks. The control mice (Con) and diabetic mice (DM) were randomly divided into four groups: vehicle-treated control group, fenofibrate-treated control group, vehicle-treated diabetic group and fenofibrate-treated diabetic group.

### 2.2 Vascular reactivity study

After anesthetizing with 2% isoflurane, the thoracic aortas and mesenteric arteries from mice were rapidly removed, placed in ice-cold Krebs-Henseleit solution, cleaned gently from adherent connective tissue and cut into approximately 2 mm length. The arteries were mounted onto a wire myograph system (model 620 M, Danish Myo Technology, Aarhus, Denmark) in oxygenated (5% CO_2_, 95% O_2_) and warmed (37 °C) Krebs solution (pH 7.4) with the following composition (mmol/L): NaCl 112, KCl 5, NaHCO_3_ 25, NaH_2_PO_4_ 1, MgCl_2_ 0.5, CaCl_2_ 2.5 and glucose 11.5. Each artery was suspended between two stainless steel wires (diameter, 40 µm) and equilibrated for 90 min at 37 °C before experiments. After that, resting tension was set according to the manufacturer's protocol and vessel viability was assessed by the response to KCl (100 mmol/L). After a wash out, cumulative concentration responses for vasoactive agents were obtained. Endothelium-dependent relaxation in response to acetylcholine (ACh, 10^−9^–10^−4^ mol/L) and endothelium-independent relaxation by sodium nitroprusside (SNP, 10^−9^–10^−4^ mol/L) were measured in aortas pre-contracted by norepinephrine (NE, 10 μmol/L). Also, a dose response to NE (10^−10^–10^−5^ mol/L) was obtained. Some segments were incubated with agonist or antagonist for 30 min before vasoreactivity measurement, include GW 6471 (10 μmol/L, PPARα inhibitor), compound C (1 μmol/L, AMPK inhibitor), 5-aminoimidazole-4 carboxamide-1-β-D- ribofuranosid (AICAR，1 μmol/L, AMPK agonist), fenofibrate (FF, 100 μmol/L, PPARα agonist), tempol (100 μmol/L, superoxide dismutase mimetic), indomethacin (100 μmol/L, cyclooxygenase inhibitor) and L-nitro-arginine-methyl-ester (L-NAME, 100 μmol/L, NOS inhibitor). For in vitro hyperglycemia experiments, aortas were incubated in normal (11.5 mmol/L) or hyperglycemic (44 mmol/L) Krebs solution for 4 h with or without fenofibrate. Then, after washing the preparation, the relaxant responses to ACh and NE were studied. All the chemicals were from Sigma-Aldrich (St Louis, MO, USA) except AICAR and GW 6471 which were purchased from ApexBio Technology (Houston，USA). Fenofibrate, AICAR, GW 6471，indomethacin and compound C were dissolved in dimethylsulfoxide (DMSO) and other drugs in distilled water. DMSO (0.1% v/v) did not modify agonist or inhibitor-induced responses.

### 2.3 Isolation and perfusion of the renal afferent arterioles

To evaluate the effect of fenofibrate on microvessels, isolation and micro-perfusion of afferent arterioles were conducted as described previously [26], [27]. In brief, kidneys were removed and sliced along the corticomedullary axis. Slices were placed in ice-cold glucose Dulbecco's modified Eagle's medium (DMEM). Afferent arterioles with attached glomeruli were micro-dissected under a stereomicroscope (SZX16, Olympus, Japan) and then transferred to a temperature-controlled chamber on the stage of an inverted microscope (Axiovert 100TV, ZEISS, Oberkochen, Germany), and perfused using a micromanipulator system with concentric holding and perfusion pipettes. Vessel viability was tested by depolarization using potassium chloride (100 mM), greater than 90% contraction was considered as viable and used in our experiments. Norepinephrine (NE: 1 × 10^−6^ mol/L) was used to preconstrict afferent arterioles before investigating dilation. Luminal diameter was measured after preconstriction and with increasing doses of ACh (10^−9^ to 10^−5^ mol/L) to test endothelium-dependent dilation in renal afferent arterioles. The arteriolar luminal diameter was calculated as the mean of seven pictures during stable tension

### 2.4 Western blot analysis

After euthanasia, the aortas were dissected and stored at − 80 °C. Frozen aortic tissue samples were homogenized with lysis buffer (Beyotime, Shanghai, China). Each protein sample was extracted from the isolated aorta of one mouse. The homogenate was centrifuged for 10 min at 13,000 g at 4 °C. Samples containing equivalent amounts of protein were loaded and separated by SDS-polyacrylamide gel electrophoresis and transferred onto polyvinylidene difluoride membranes. Membranes were blocked by 5% fat-free milk powder and immunodetected with specific primary antibodies to β-actin, eNOS, p-LKB1 (Ser334), LKB1, p-AMPK (Thr172), AMPK, COX-2, NF-κB p65 (Ser 468) (Abcam, Cambridge, UK); p-eNOS (Ser1177) (Cell Signaling Technology, Beverly, MA, USA), Histone H3 (Beyotime Biotechnology, Shanghai, China) and second horseradish peroxidase-labeled IgG anti-rabbit (or mouse) antibody (Beyotime Biotechnology, Shanghai, China). Immunoreactivity was incubated by enhanced chemiluminescence (Beyotime Biotechnology, Shanghai, China) and visualized in an automated imaging analysis system (Tanon 5200 Multi, Tanon Science & Technology Inc, Shanghai, China).

### 2.5 Biochemical markers of renal function and blood lipid

Animals were anesthetized with 2% isoflurane. Blood was sampled from the inferior vena cava and centrifuged at 3000 rpm, 4 °C for 15 min. Serum was stored at − 80 °C until analysis. Serum creatinine, blood urea nitrogen and blood lipids were measured using commercial kits (Roche, Germany).

### 2.6 Measurement of hydrogen peroxide

After euthanasia, the aortas were dissected and stored at − 80 °C. Aortic hydrogen peroxide (H_2_O_2_) was measured according to the manufacturer's instructions of H_2_O_2_ analysis kit (Beyotime Biotechnology, Shanghai, China) as described previously [27]. Briefly, frozen aortic tissue samples were homogenized. The supernatants of homogenized aortas were collected on ice and centrifuged for 10 min at 13,000 g at 4 °C. Subsequently, the aortic concentration of H_2_O_2_ were measured by incubation of supernatants with H_2_O_2_ test solutions for 30 min at room temperature and detection of the absorbance at 560 nm.

### 2.7 Measurement of enzymatic activity

The supernatants were sampled from homogenized aortas for analysis of catalase activity and superoxide dismutase activity (SOD) levels according to the manufacturer's instructions of Catalase Assay Kit and SOD Assay Kit (Beyotime Biotechnology, Shanghai, China) as described previously [27].

### 2.8 Measurement of nitric oxide

Levels of the nitric oxide (NO) were determined in the serum and aortic tissues. A nitrite oxide detection kit (Beyotime, Shanghai, China) was used according to the manufacturer's instructions.

### 2.9 Measurement of TxA2 and PGE2

Levels of the PGE2 and TxA2 were determined in the serum using a kit (Yeyuan, Shanghai, China) according to instructions provided by the manufacturer.

### 2.10 Statistical analysis

Data are present as Mean±SEM. One-way ANOVA followed by a Bonferroni multiple comparison test was performed when multiple groups were compared. Two-way repeated measures ANOVA was used to determine the vascular reactivity differences among groups. All statistical calculations were made using GraphPad Prism Software 6.0. Statistical significance was defined as p < 0.05.

## 3 Results

### 3.1 Fenofibrate administration ameliorates renal dysfunction and reduces blood lipids in diabetic mice

Body weights of mice were significantly lower and blood glucose levels were higher in the diabetic mice (DM) compared with the control mice (Con) (Table 1). Fenofibrate treatment did not affect body weights or blood glucose in diabetic mice. However, fenofibrate treatment decreased the levels of plasma triacylglycerol compared with vehicle-treated DM (4.6 ± 2.2 versus 1.8 ± 0.8 mmol/L, p < 0.001), tended to decrease total cholesterol and increase high-density lipoprotein-cholesterol. Also, fenofibrate treatment decreased serum creatinine compared with vehicle-treated DM (26 ± 2 versus 22 ± 0.9 μmol/L, p < 0.01) and showed a trend towards lower serum urea nitrogen (Table 1).

**Table 1:** Fenofibrate administration ameliorates renal dysfunction and blood lipids in diabetic mice. Characteristics of vehicle and fenofibrate-treated control mice (Con) and diabetic mice (DM).

**Fenofibrate**	**Con**		**DM**	
	**−**	**+**	**−**	**+**
Body weight (g)	25.1 ± 1.5	26.5 ± 1.1	20.1 ± 0.9^***^	19.8 ± 1.3
Blood glucose (mmol/L)	6.6 ± 0.8	6.5 ± 0.7	22.6 ± 1.9^***^	21.7 ± 1.8
Triglycerides (mmol/L)	1.2 ± 0.4	1.4 ± 0.1	4.6 ± 2.2^***^	1.8 ± 0.8^###^
Cholesterol (mmol/L)	2.4 ± 0.1	2.6 ± 0.3	2.6 ± 0.5	2.5 ± 0.7
HDL-cholesterol (mmol/L)	1.9 ± 0.2	1.9 ± 0.3	1.8 ± 0.2	2 ± 0.5
Serum creatinine (μmol/L)	12 ± 2	13 ± 1.9	26 ± 2^***^	22 ± 0.9^##^
Serum urea nitrogen (mmol/L)	9.8 ± 1.6	11 ± 0.6	14 ± 1^**^	12 ± 1.7

Data are mean±SEM. ***p < 0.001 vs Con, ^###^p < 0.001 vs vehicle-treated DM. n = 6.

### 3.2 Fenofibrate treatment improves vascular dysfunction of aortas in diabetic mice

**Fig. 1:**
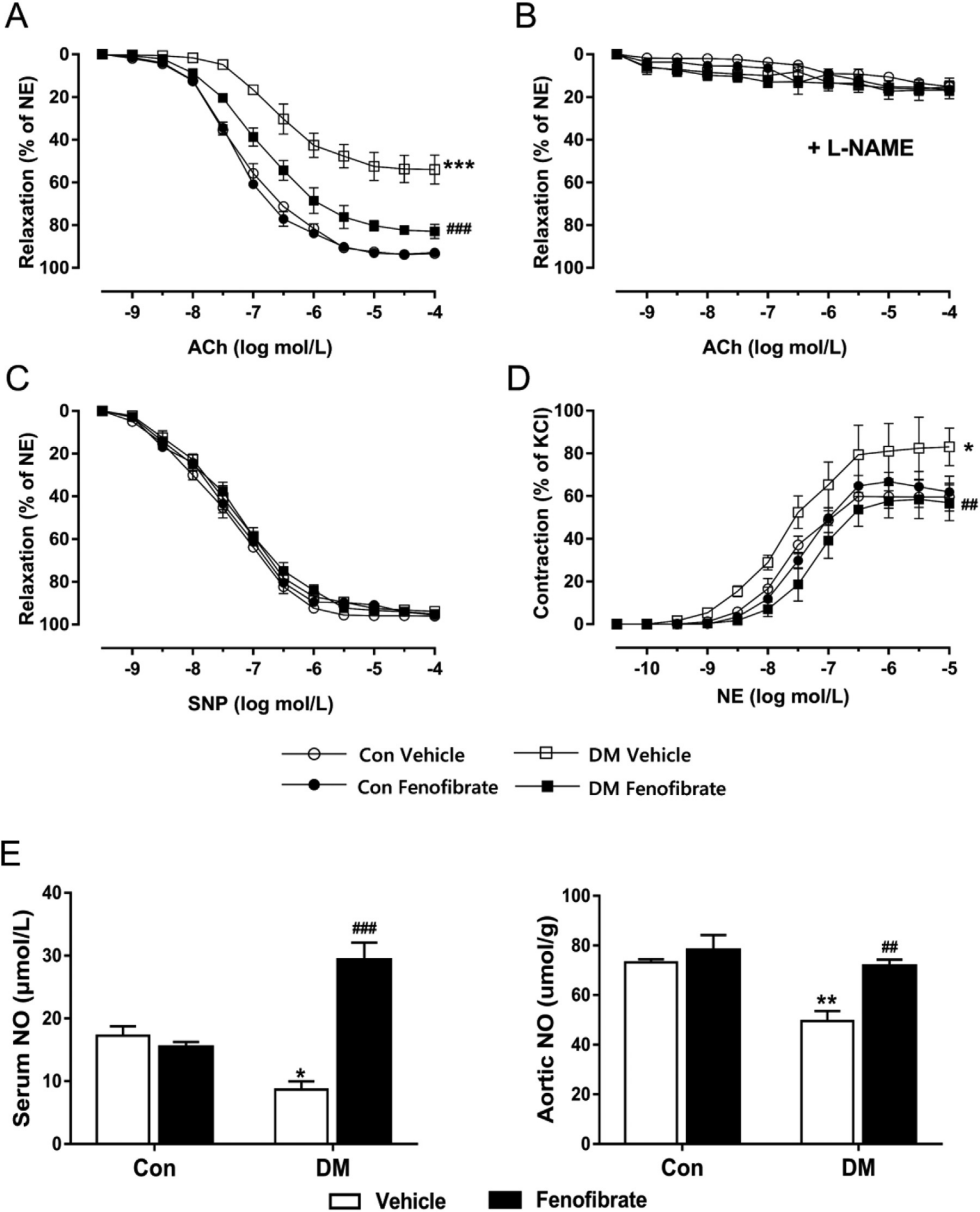
Fenofibrate treatment improves vasodilatory and vasoconstrictive dysfunction in aorta from diabetic mice. A, Endothelium-dependent relaxation by acetylcholine (ACh) in aortas from vehicle and fenofibrate-treated control mice (Con) and diabetic mice (DM). B, Vasodilatory responses to ACh with 30 min preincubation of L-NAME (100 μmol/L, NOS inhibitor) treatment in all groups. C, Endothelium-independent relaxation to sodium nitroprusside (SNP) in aortas from vehicle and fenofibrate-treated Con and DM. D, Vascular contractility by norepinephrine (NE) in aortas from vehicle and fenofibrate-treated Con and DM. E, Levels of NO in serum and aortic tissues from vehicle and fenofibrate-treated Con and DM. Data are mean±SEM. ***p < 0.001 vs Con, ^###^p < 0.001 vs vehicle-treated DM. n = 6.

Eight-week fenofibrate administration markedly improved endothelium-dependent relaxation (EDR) by acetylcholine in DM aortas without affecting responses in control mice (Fig. 1A, p < 0.001). The NO synthase inhibitor L-NAME inhibited acetylcholine-induced relaxation in all four groups (Fig. 1B). Endothelium-independent relaxation by the NO donor sodium nitroprusside was similar in all four groups (Fig. 1C). Also, fenofibrate treatment improved vascular contractile responses to noradrenaline in DM aortas compared with vehicle-treated DM (Fig. 1D, p < 0.001). The NO concentrations were greater in eight-week fenofibrate-treated DM compared with vehicle-treated DM (Fig. 1E, serum NO, 29.3 ± 5.6 versus 8.7 ± 2.6 μmol/L; aortic NO, 71.7 ± 5.2 versus 49.5 ± 8.2 μmol/g, p < 0.01). This matched with the increased vasodilation in the aortas from fenofibrate-treated DM.

### 3.3 Fenofibrate treatment improves vascular dysfunction of microvessels in diabetic mice

**Fig. 2:**
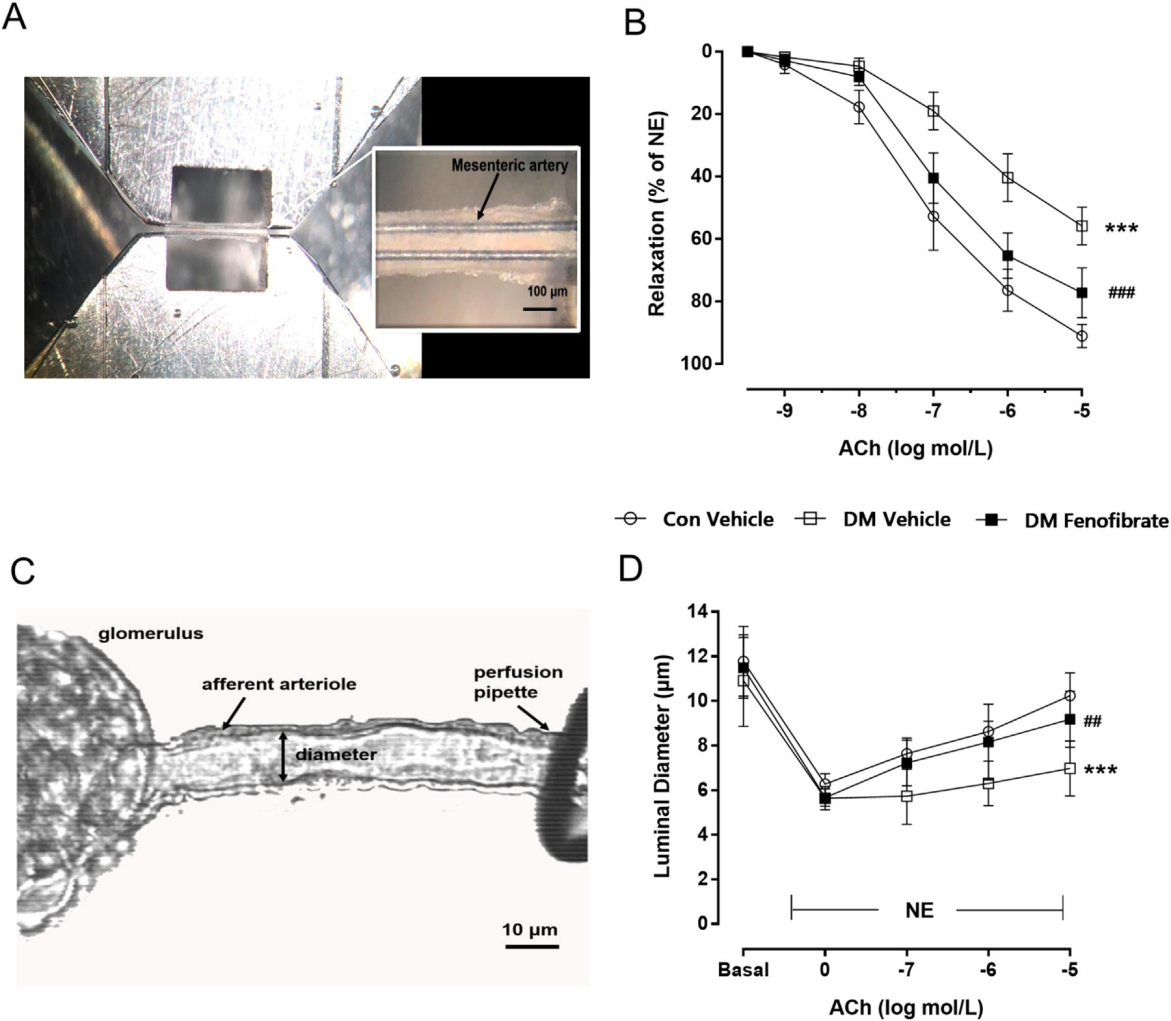
Effect of fenofibrate on mesenteric artery and isolated renal afferent arteriole relaxation. A, Mesenteric artery mounted on a myograph with vascular tone recorded. The bar is 100 µm. B. Effect of fenofibrate on mesenteric artery relaxation. C, Isolated perfused renal afferent arteriole mounted on a perfusion pipette with diameter recorded. The bar is 10 µm. D, Effect of fenofibrate on isolated renal afferent arteriole relaxation. ***p < 0.001 vs Con, ^###^p < 0.001 vs vehicle-treated DM. n = 5.

To evaluate the effect of eight-week fenofibrate administration on micro vessels, vascular reactivity of the mesenteric artery and renal afferent arterioles were conducted. Results showed that eight-week fenofibrate administration markedly improved EDR in DM mesenteric artery and afferent arteriole without affecting responses in control mice (Fig. 2, p < 0.001). This matched with the increased vasodilation in the aortas from fenofibrate-treated DM.

### 3.4 Effect of short time treatment of fenofibrate on mesenteric artery relaxation

**Fig. 3:**
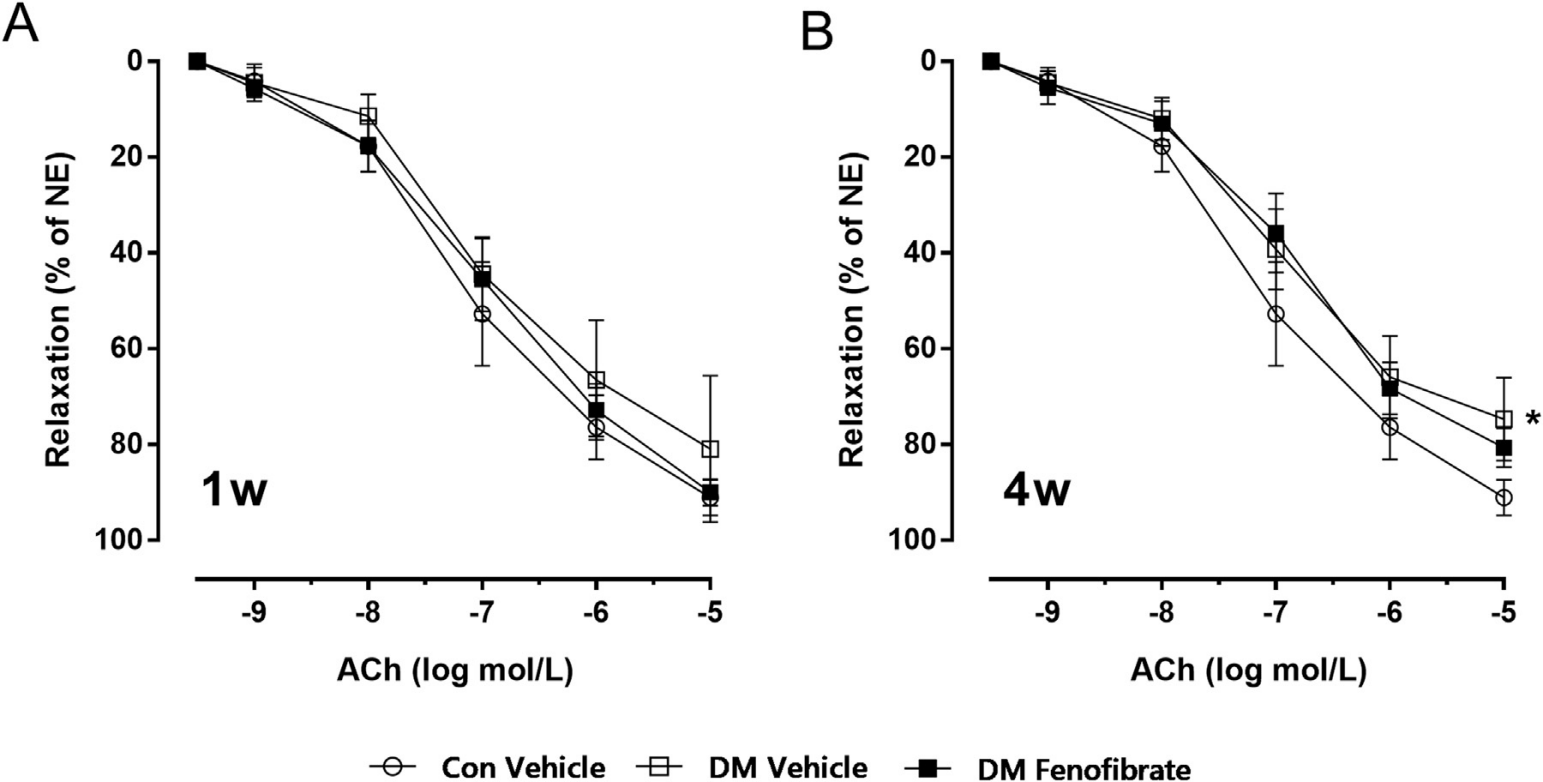
Effect of short time treatment of fenofibrate on mesenteric artery relaxation. A, Effect of one-week treatment of fenofibrate on mesenteric artery relaxation. B, Effect of four-week treatment of fenofibrate on mesenteric artery relaxation. *p < 0.05 vs Con. n = 5.

To evaluate the vascular functions during the time course of streptozotocin-induced diabetes under fenofibrate treatment, we conducted vascular experiments after short time treatment with fenofibrate and monitored mesenteric artery relaxation. Our results showed that one-week or four-week treatment of fenofibrate were not sufficient to improve vascular function in diabetic mice, but eight-week fenofibrate treatment improved vascular function. (Fig. 3, p < 0.001)

### 3.5 Fenofibrate improves endothelial function via the PPAR/LKB1/AMPK/eNOS pathway in diabetic mice

**Fig. 4:**
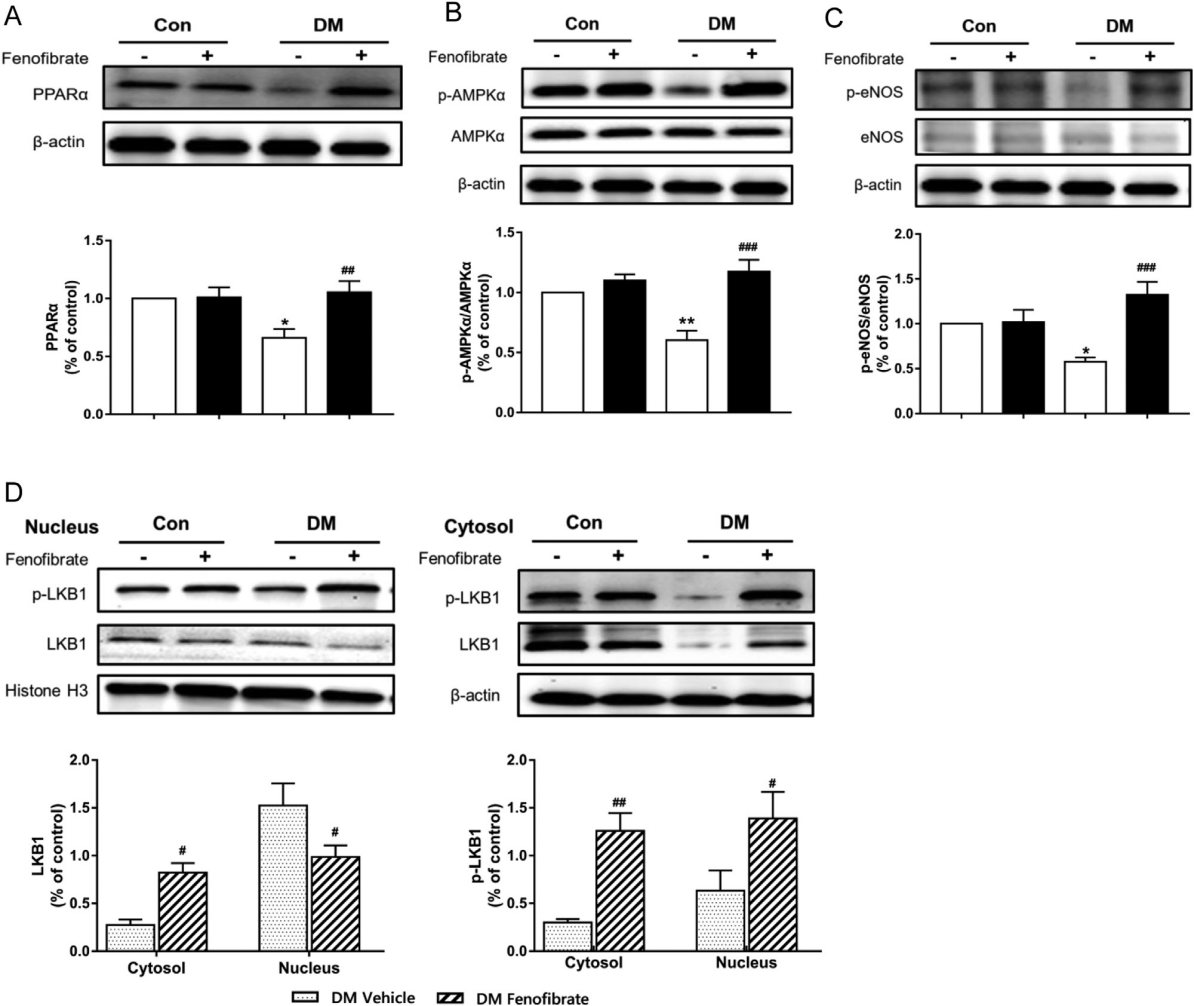
Endothelial function improvement by fenofibrate is through normalizing eNOS activity in diabetic mice. A, Protein expression of PPARα in aortas from vehicle and fenofibrate-treated control mice (Con) and diabetic mice (DM). B, Protein expression of LKB1 and phosphorylations of LKB1 in nucleus and cytosol of aortas from vehicle and fenofibrate-treated Con and DM. Phosphorylation of AMPKα (C) and eNOS (D) in aortas from vehicle and fenofibrate-treated Con and DM. ***p < 0.001 vs Con, ^###^p < 0.001 vs vehicle-treated DM. n = 4.

Compared with the vehicle-treated DM, phosphorylation of eNOS (Ser 1177) was 2.4 folder higher in aortas from fenofibrate-treated DM (Fig. 4D, p < 0.001). This matched with an increased NO concentration in the aortas from fenofibrate-treated DM (Fig. 4E). To further investigate the underlying mechanism, we evaluated PPARα expression and phosphorylation of LKB1 and AMPKα in aortas. Western blots showed that protein expression of PPARα and AMPKα phosphorylation were decreased in vehicle-treated DM, whereas fenofibrate treatment upregulated both. Compared with the vehicle-treated DM, the phosphorylation of LKB1 was increased both in the nucleus and cytosol in aortas from fenofibrate-treated DM, while protein expression of LKB1 was markedly reduced in the nucleus and increased in the cytosol from fenofibrate-treated DM. This indicates that LKB1 transfers from the nucleus to the cytosol and then activates AMPK in fenofibrate-treated DM.

### 3.6 Protective effect of fenofibrate is abolished by PPARα antagonist and AMPKα inhibitor

**Fig. 5:**
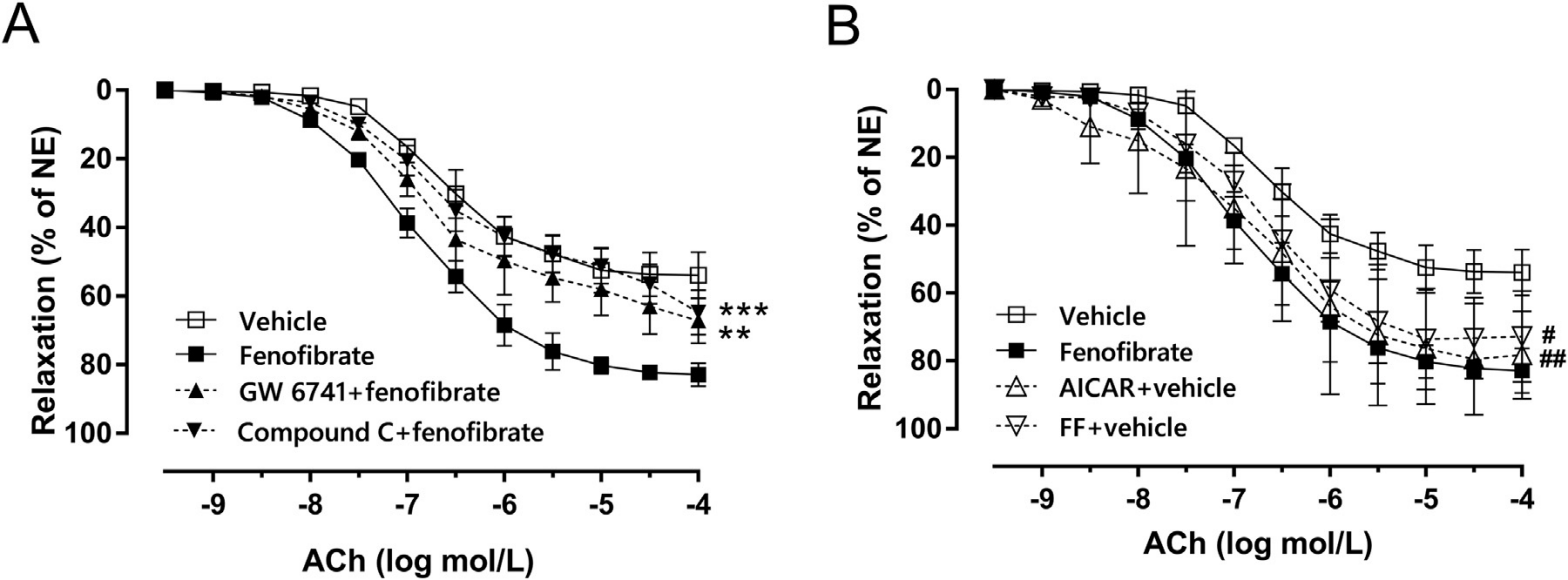
Protective effect of fenofibrate is abolished by PPARα antagonist and AMPKα inhibitor A, Effect of 30-min preincubation with GW 6471 (10 μmol/L, PPARα inhibitor) and Compound C (1 μmol/L, AMPK inhibitor) on endothelium-dependent relaxation (EDR) in aorta from fenofibrate-treated DM. B, Effects of 30-min preincubation with FF (100 μmol/L, PPARα agonist) and AICAR (1 μmol/L, AMPK agonist) on EDR in aorta from vehicle-treated DM. Data are mean±SEM. *** p < 0.001 vs fenofibrate-treated DM, ^###^ p < 0.001 vs vehicle-treated DM. n = 6.

To understand how activation of these signals contributes to vascular reactivity, we used two selective inhibitors to incubate aortas from fenofibrate-treated DM: GW6471 inhibits PPARα and compound C inhibits AMPKα phosphorylation. After incubation with either drug, the vascular protection of fenofibrate was reversed (Fig. 5A, p < 0.01). Furthermore, we used two selective agonists to incubate aortas from vehicle-treated DM: AICAR activates AMPK and FF activates PPARα. We found that both drugs increased vasodilation of aortas (Fig. 5B, p < 0.01), suggesting a direct modulatory effect of fenofibrate on vascular tone via PPARα- and AMPKα-related pathways in DM. Also, this indicates that the effect of fenofibrate on vascular function is independent of its hypolipemic action.

### 3.7 Diabetes-associated oxidative stress is attenuated by fenofibrate treatment

**Fig. 6:**
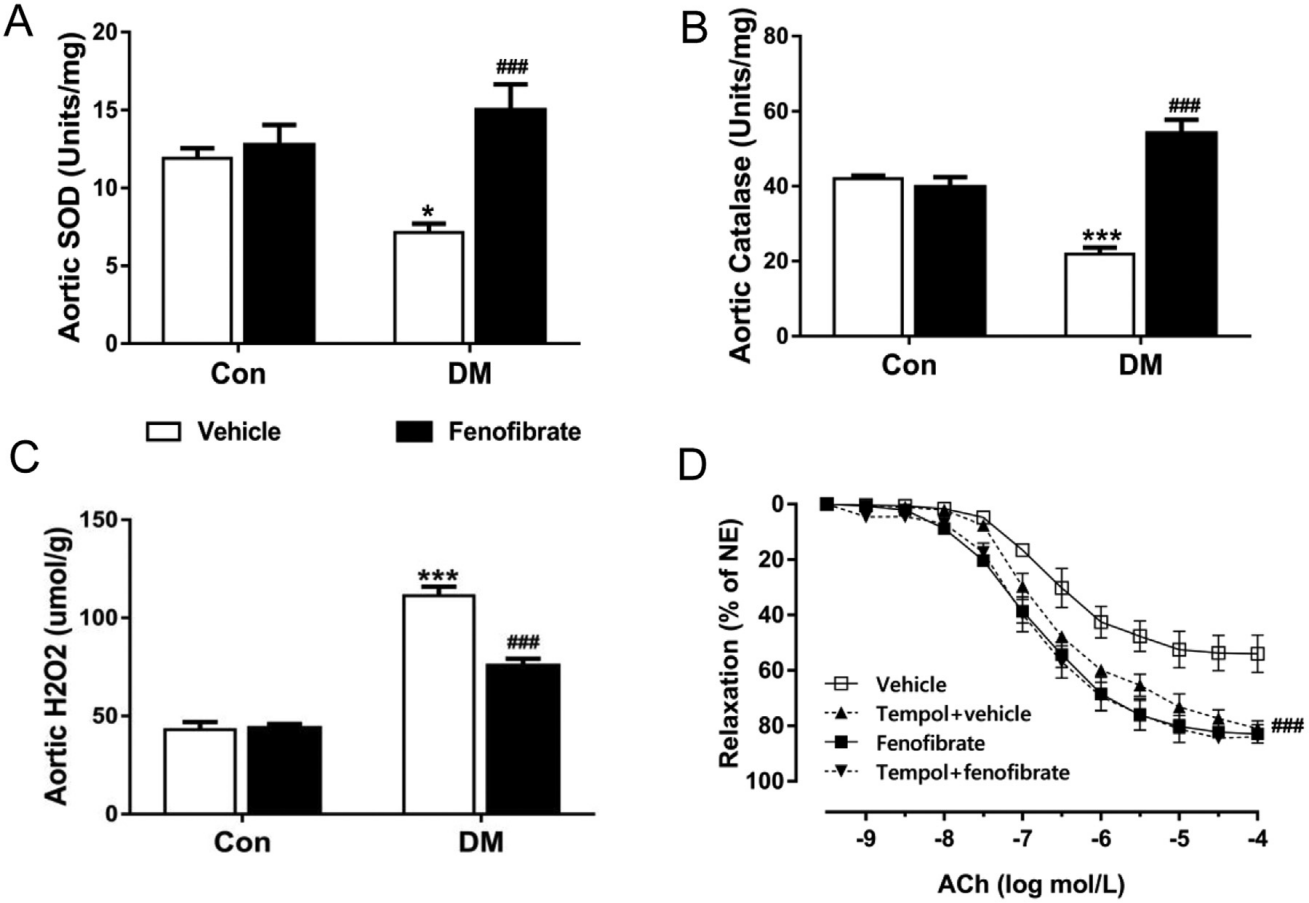
Diabetes-associated oxidative stress is attenuated by fenofibrate treatment. A, Levels of superoxide dismutase (SOD) in aortic tissues from vehicle and fenofibrate-treated control mice (Con) and diabetic mice (DM). B, Levels of catalase in aortas from vehicle and fenofibrate-treated Con and DM. C, Levels of hydrogen peroxide (H2O2) in aortas from vehicle and fenofibrate-treated Con and DM. D, Effect of 30-min preincubation with tempol (100μmol/L, superoxide dismutase mimetic) on endothelium dependent relaxation in aortas from vehicle and fenofibrate-treated DM. Data are mean±SEM. ***p < 0.001 vs Con, ^###^p < 0.001 vs vehicle-treated DM. n = 6.

Since diabetes is associated with excessive superoxide production that may reduce NO bioavailability, we tested levels of oxidative stress in aortic tissues. Levels of superoxide dismutase (SOD) and catalase were both reduced and hydrogen peroxide (H_2_O_2_) was increased in vehicle-treated DM. However, all of these changes were reversed by fenofibrate treatment (Fig. 6A to C, p < 0.001). Also, incubation with the permeable superoxide mimetic tempol increased the vasodilatory response to acetylcholine in vehicle-treated DM (Fig. 6D), but failed to change acetylcholine responses in fenofibrate-treated DM, further supporting the inhibitory role of fenofibrate on oxidative stress in diabetes.

### 3.8 Fenofibrate treatment normalizes vasoconstriction via the NF-κB/COX-2 pathway in diabetic mice

**Fig. 7:**
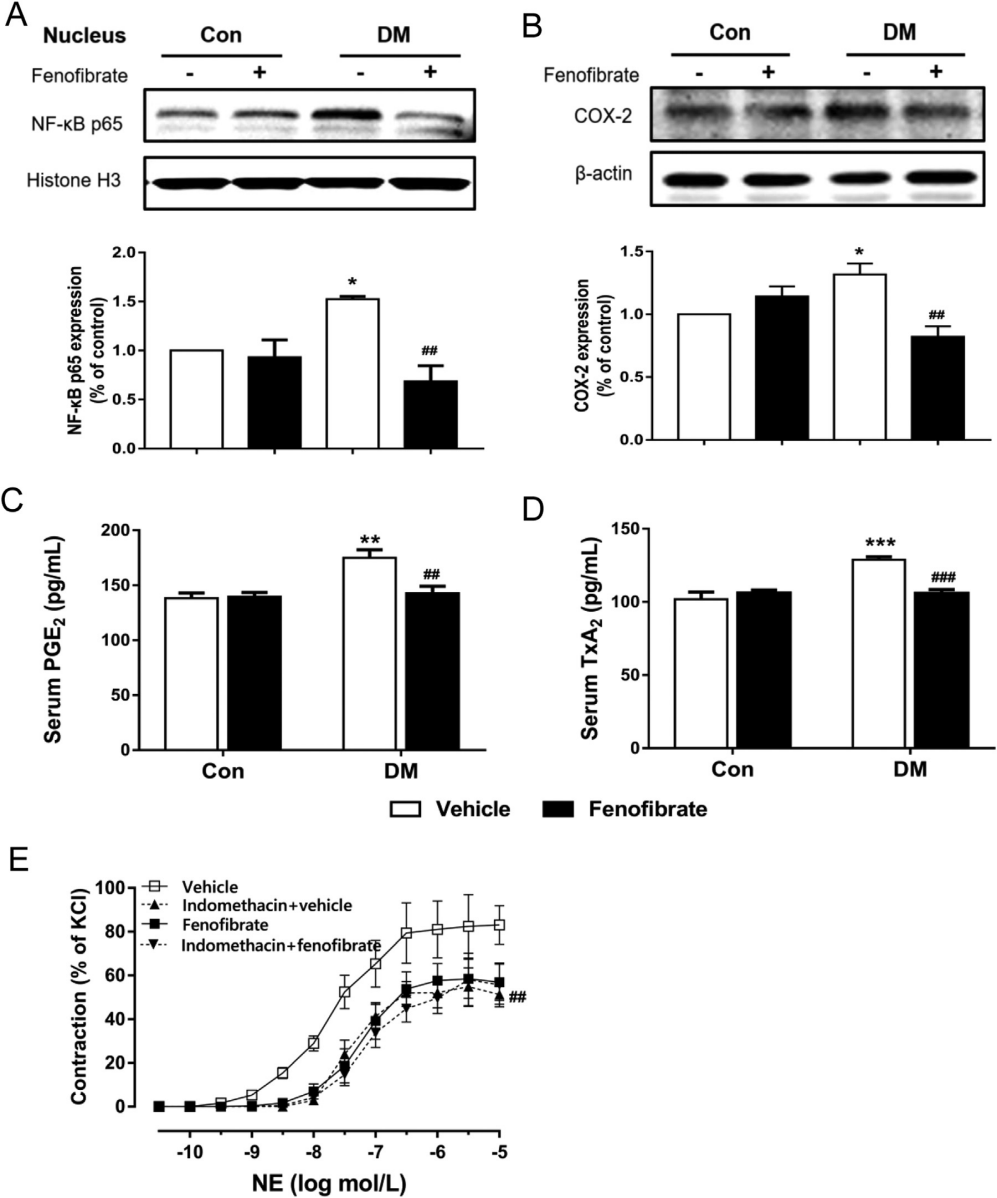
Fenofibrate treatment normalizes vasoconstriction via the NF-κB/COX-2 pathway in diabetic mice. A, Protein expression of NF-κB p65 in the nucleus of aortas from vehicle and fenofibrate-treated control mice (Con) and diabetic mice (DM). B, Protein expression of COX-2 in aortas from vehicle and fenofibrate-treated Con and DM. C and D, Levels of prostaglandin E2 (PGE2) and thromboxanes (TxA2) in serum from vehicle and fenofibrate-treated Con and DM. E, Effect of 30-min preincubation with indomethacin (100μmol/L, cyclooxygenase inhibitor) on endothelium-dependent vasoconstriction in aortas from vehicle and fenofibrate-treated DM. Data are mean±SEM. *p < 0.05 vs Con, ^##^p < 0.01 vs vehicle-treated DM. n = 5.

Compared with the vehicle-treated DM, fenofibrate administration decreased COX-2 and NF-κB p65 proteins in the aorta (Fig. 7A and B, p < 0.01). Levels of TxA2 and PGE2 in serum were both increased in vehicle-treated DM and were reversed by fenofibrate treatment (Fig. 7C and D, p < 0.01). Also, incubation of aortas with indomethacin to inhibit cyclooxygenase restored the enhanced NE-mediated contraction in vehicle-treated DM, but did not show any effect in aortas from fenofibrate-treated DM (Fig. 7E). This indicates a modulatory effect of fenofibrate on vasocontractility via the COX-2 related pathway. Therefore, improvement of vascular contractility by fenofibrate treatment can be largely attributed to suppression of the vasoconstrictor prostaglandin via inhibition of the NF-κB/COX-2 pathway.

### 3.9 Effect of fenofibrate on vascular reactivity altered by high glucose concentrations in vitro

**Fig. 8:**
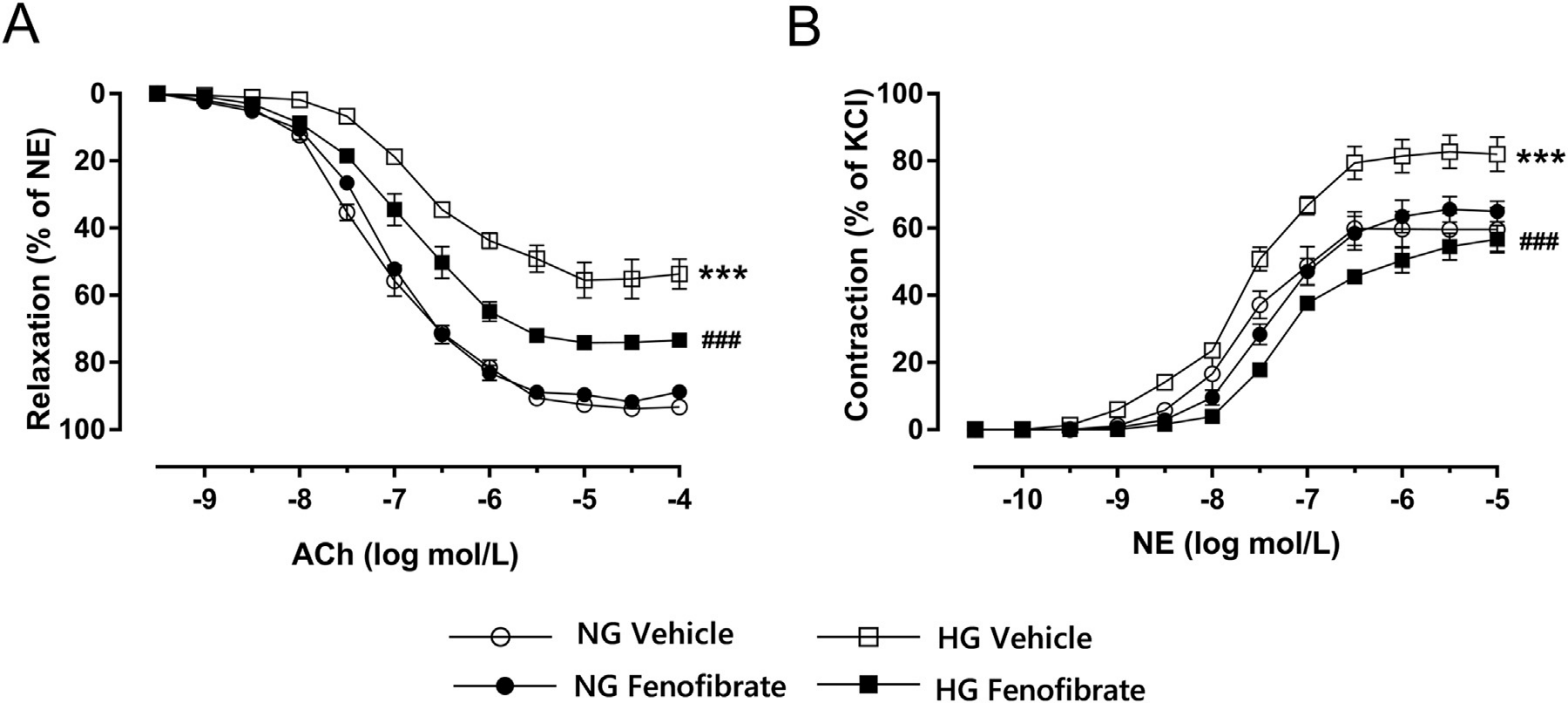
Role of fenofibrate in vascular reactivity induced by high glucose concentrations in vitro. A, Endothelium-dependent relaxation by acetylcholine (ACh) in aortas after 4 h incubation with normal (11.5 mmol/L, NG) or high (44 mmol/L, HG) glucose medium with or without fenofibrate (100 μmol/L) in vitro. B, Vascular contractility by norepinephrine (NE) in aortas after 4 h of incubation with NG or HG with or without fenofibrate (100 μmol/L) in vitro. Data are mean±SEM. ***p < 0.001 vs NG, ^###^p < 0.001 vs HG. n = 5.

ACh-induced vasodilation was markedly impaired and the contractile responses to NE were greater in control aortas incubated with hyperglycemic concentrations, compared with normal concentrations of glucose. Incubation with fenofibrate improved vascular relaxation and constriction under high glucose concentration, while it did not affect the vascular response in normal glucose concentration (Fig. 8A and B, p < 0.001).

## 4 Discussion

The present study was designed to evaluate the effects of fenofibrate on endothelial function in diabetes and explore possible signaling mechanisms involved. We used a mouse model of STZ-induced DM and investigated effects of an eight-week treatment with fenofibrate (100 mg/kg/d) on endothelial dysfunction. Our results show that fenofibrate treatment reduced renal damage markers and plasma triglyceride levels, while there was no significant change in blood glucose and body weight between vehicle and fenofibrate-treated DM. Eight-week fenofibrate treatment improved endothelium dependent relaxation in macro- and microvessels, increased NO levels and inhibited oxidative stress. Similarly, incubating aortas from wild type mice with a hyperglycemic concentration exhibited a remarkably weaker vascular relaxation, whereas fenofibrate treatment reversed this change. The aorta vasodilation by fenofibrate treatment was reversed by a PPARα and by an AMPKα inhibitor. Western blot results showed that fenofibrate treatment elevated PPARα expression, subsequently induced LKB1 translocation from the nucleus to the cytoplasm to activate AMPKα and thus activated eNOS. The vascular relaxation effect of fenofibrate most likely is through modulation of the PPAR/LKB1/AMPK/eNOS pathway to increase production of NO and suppress oxidative stress. We found that fenofibrate treatment reduced levels of the vasoconstrictor prostaglandin and decreased the protein expressions of NF-κB p65 and cyclooxygenase 2 in aortas. Incubation with indomethacin improved aortic contractility in diabetic mice. The vascular contractility effect of fenofibrate most likely is via inhibition of the NF-κB/COX-2 pathway to reduce vasoconstrictor prostaglandin. Overall, our results show that fenofibrate treatment improved the vascular endothelial function by balancing endothelium-dependent relaxation and contractility in diabetic mice.

Previous clinical studies have noted the importance of vascular complications of diabetes. Both the FIELD and ACCORD study reported that fenofibrate treatment in type 2 diabetic patients reduces the need for laser treatment of diabetic retinopathy, delayed the progression of diabetic nephropathy and reduced nonfatal coronary events [10], [11]. The mechanisms of fenofibrate action responsible for these benefits were not apparent. Endothelial dysfunction is the most likely culprit for vascular complications of diabetes mellitus. A proposed core event are diminished amounts of NO due to reduced expression or activity of eNOS [28]. We found that fenofibrate treatment stimulated NO production and increased phosphorylation of eNOS in arteries from diabetic mice. Also, endothelium-dependent relaxation is impaired in aortas from diabetic mice and this vascular impairment is corrected by treatment with fenofibrate. Previous studies showed that eNOS could be phosphorylated on serine 1177 by AMPK [29], [30]. We found that fenofibrate administration increased the protein expression of PPARα and AMPKα phosphorylation in aortas from diabetic mice. This result was consistent with previous cell experiments reporting that fenofibrate has a role in AMPKα activation [19], [20], but the mechanism by which fenofibrate activates AMPK is poorly defined. Interestingly, a potentially important observation in our study is that fenofibrate administration upregulated LKB1 by transferring from the nucleus to the cytoplasm and increased phosphorylation of LKB1, thus activating AMPK in the cytosol. Previous studies reported that LKB1 has many biological pathways and deletion of LKB1 has a directly negative affect on AMPK [31]. Therefore, we propose that fenofibrate activates PPARα, induces LKB1 transferring from the nucleus to the cytosol, upregulates LKB1 phosphorylation, activates AMPKα and increases eNOS phosphorylation. Our in vitro vascular experiments further confirm our hypothesis. Preincubation of aortas with GW 6471 (a PPARα inhibitor) and Compound C (an AMPK inhibitor) from fenofibrate-treated DM did reverse the improvement in endothelium-dependent relaxation. Also, preincubation of aortas with FF (a PPARα agonist) or AICAR (an AMPK agonist) restored acetylcholine-induced vasodilation in vehicle-treated DM, but did not affect aorta from fenofibrate-treated DM. This strongly suggests a direct modulatory effect of fenofibrate on vascular tone via PPARα- and AMPKα-related pathways in diabetic mice. Also, incubating aortas with fenofibrate improved endothelial-dependent relaxation under high glucose conditions suggesting that fenofibrate has positive effects on vascular function independent of its hypolipemic action. Together, our data show that fenofibrate normalizes endothelium-dependent relaxation in aortas via the activation of PPAR/LKB1/AMPK/eNOS pathway signals to increase NO production in diabetic mice.

Amongst the underlying mechanisms in the diabetes-related macro- and microvascular complications, except for impaired eNOS derived NO production, oxidative stress also plays a vital role [3], [32], which reducing NO bioavailability. We found that levels of SOD and catalase were increased and hydrogen peroxide was decreased in aortic tissues in fenofibrate-treated diabetic mice compared with vehicle-treated diabetic mice, which indicating an inhibition of oxidative stress by fenofibrate in diabetes. It is thought that SOD promotes the conversion of superoxide into oxygen and H_2_O_2_, which is then rapidly decomposed by the enzyme catalase to oxygen and water. However, H_2_O_2_ level was higher than controls, in spite of either low level of SOD in vehicle-treated diabetic mice or higher level of catalase in fenofibrate-treated diabetic mice. This discrepancy may be attributed by oxidases in peroxisomes, which are other sources of H_2_O_2_ [33], [34]. Indeed, the alterations in peroxisomal redox metabolism have been associated with the etiology and progression of diabetes [35]. Previously, it has been shown that AMPK plays a vital role in regulating mitochondrial function and reduces intracellular ROS levels [14], [36] through increased expression or activation of LKB1 [31]. Therefore, we conclude that activation of PPAR/LKB1/AMPK/eNOS signaling by fenofibrate may also relate to the inhibition of oxidative stress and these effects together improve endothelial function in diabetes.

Excessive superoxide production results in formation of peroxynitrite, a potent oxidant agent [37], [38]. Increased peroxynitrite generation is believed to contribute to numerous pathologies, including chronic heart failure, diabetes, stroke and local inflammatory diseases [39]. Peroxynitrite has also been implicated in the loss of vascular endothelium-dependent responses [40]. Since peroxynitrite generation is linked closely to the production of superoxide, inhibition of oxidative stress by fenofibrate treatment thus could reduce peroxynitrite production and prevent peroxynitrite induced cytotoxicity and vascular injury.

In diabetic animal models, COX-2 expression was found upregulated and COX-2-derived prostaglandins were shown to induce abnormal vasoconstrictor responses [41], [42]. The present finding is consistent with these previous reports. Vascular constriction, protein expression of COX-2, and levels of TxA2 and PGE2 were all increased in diabetic mice. However, fenofibrate administration reversed these changes. We also found that nuclear NF-κB p65 protein in aortas decreased in fenofibrate-treated diabetic mice. Incubation of aortas with indomethacin to inhibit the cyclooxygenase, restored enhanced NE-mediated contraction in vehicle-treated DM, but did not affect aortas from fenofibrate-treated DM, which indicates that fenofibrate plays a modulatory effect on vasoconstrictive tone via inhibiting the NF-κB/COX-2 pathway. In accordance with our present results, previous studies have demonstrated that inhibition of NF-κB activity normalized TxA2 release in aortas from diabetic rats [43] and have shown that enhanced NF-κB activity impairs vascular function through COX-2-dependent mechanism in diabetic arteries [44]. Furthermore, prior studies that have noted that COX-2-derived TxA2 release could be responsible for the decreased NO bioavailability in aortas from aged rats [45]. Similarly, production of NO was augmented in thromboxane prostanoid knockout mice [46]. The studies suggested that COX-2 has an opposing regulatory effect on the release of NO. Therefore, as discussed above, normalization of NO levels by fenofibrate treatment of diabetic mice, may partly result from the inhibition of the NF-κB/COX-2 signal by fenofibrate. Thus, we propose an important role of fenofibrate in regulating the COX-derived constrictor prostaglandins in diabetic mice.

To the best of our knowledge, we are the first to reveal the balance of vascular endothelial-dependent relaxation and constriction in evaluating the effect of fenofibrate in type I diabetic mice. A novel finding in our study is that we found that fenofibrate activates AMPK by increasing LKB1 phosphorylation and by inducing LKB1 translocation from the nucleus to the cytoplasm and thus activate AMPK. Another interesting observation is that endothelial protection by fenofibrate is partly driven by the inhibition of a COX-related pathway. As a potential weakness of our current study, we did not use PPARα, AMPKα or COX knockout mice, but we conducted the vascular experiments with pharmacologic reagents i.e. agonists and inhibitors, which demonstrate their role in the vascular endothelium.

In summary, we show that chronic fenofibrate administration normalizes endothelial function by balancing endothelial-dependent relaxation and constriction in diabetic mice. The effect of increased endothelium-dependent relaxation is via activating the PPAR/LKB1/AMPK/eNOS pathway, thus increasing endogenous NO generation and preventing oxidative stress. Contractile responses were related to the inhibition of NF-κB/COX-2 pathway and thus suppressing vasoconstrictor prostaglandin. Our findings give further insights into the mechanisms underlying the vascular protective effect of fenofibrate in diabetic endothelial function, suggesting a potential advantage of intervention with fenofibrate as a therapeutic approach to diabetes-related vascular complications.

## Funding

This study was supported by research grants to En Yin Lai from the National Natural Science Foundation of China (31471100 and 31671193), and the Key research development program of Ningxia Hui autonomous regional projects (2018YBZD0557).

## References

[1] Flammer A.J., Anderson T., Celermajer D.S., Creager M.A., Deanfield J., Ganz P., Hamburg N.M., Luscher T.F., Shechter M., Taddei S., Vita J.A., Lerman A. (2012). The assessment of endothelial function: from research into clinical practice. Circulation.

[2] Favero G., Paganelli C., Buffoli B., Rodella L.F., Rezzani R. (2014). Endothelium and its alterations in cardiovascular diseases: life style intervention. Biomed. Res. Int..

[3] De Vriese A.S., Verbeuren T.J., Van de Voorde J., Lameire N.H., Vanhoutte P.M. (2000). Endothelial dysfunction in diabetes. Br. J. Pharmacol..

[4] Ding H., Triggle C.R. (2010). Endothelial dysfunction in diabetes: multiple targets for treatment. Pflug. Arch..

[5] Jay D., Hitomi H., Griendling K.K. (2006). Oxidative stress and diabetic cardiovascular complications. Free Radic. Biol. Med..

[6] Tang E.H., Vanhoutte P.M. (2009). Prostanoids and reactive oxygen species: team players in endothelium-dependent contractions. Pharmacol. Ther..

[7] Vanhoutte P.M., Shimokawa H., Tang E.H., Feletou M. (2009). Endothelial dysfunction and vascular disease. Acta Physiol. (Oxf).

[8] Keating G.M. (2011). Fenofibrate: a review of its lipid-modifying effects in dyslipidemia and its vascular effects in type 2 diabetes mellitus. Am. J. Cardiovasc. Drugs.

[9] Rosenson R.S. (2008). Fenofibrate: treatment of hyperlipidemia and beyond. Expert Rev. Cardiovasc. Ther..

[10] Keech A., Simes R.J., Barter P., Best J., Scott R., Taskinen M.R., Forder P., Pillai A., Davis T., Glasziou P., Drury P., Kesaniemi Y.A., Sullivan D., Hunt D., Colman P., d'Emden M., Whiting M., Ehnholm C., Laakso M., F.s. investigators (2005). Effects of long-term fenofibrate therapy on cardiovascular events in 9795 people with type 2 diabetes mellitus (the FIELD study): randomised controlled trial. Lancet.

[11] Keech A.C., Mitchell P., Summanen P.A., O'Day J., Davis T.M., Moffitt M.S., Taskinen M.R., Simes R.J., Tse D., Williamson E., Merrifield A., Laatikainen L.T., d'Emden M.C., Crimet D.C., O'Connell R.L., Colman P.G., F.s. investigators (2007). Effect of fenofibrate on the need for laser treatment for diabetic retinopathy (FIELD study): a randomised controlled trial. Lancet.

[12] Group A.S., Group A.E.S., Chew E.Y., Ambrosius W.T., Davis M.D., Danis R.P., Gangaputra S., Greven C.M., Hubbard L., Esser B.A., Lovato J.F., Perdue L.H., Goff D.C., Cushman W.C., Ginsberg H.N., Elam M.B., Genuth S., Gerstein H.C., Schubart U., Fine L.J. (2010). Effects of medical therapies on retinopathy progression in type 2 diabetes. N. Engl. J. Med..

[13] Lee W.H., Kim S.G. (2010). AMPK-dependent metabolic regulation by PPAR agonists. PPAR Res..

[14] Ceolotto G., Gallo A., Papparella I., Franco L., Murphy E., Iori E., Pagnin E., Fadini G.P., Albiero M., Semplicini A., Avogaro A. (2007). Rosiglitazone reduces glucose-induced oxidative stress mediated by NAD(P)H oxidase via AMPK-dependent mechanism. Arterioscler. Thromb. Vasc. Biol..

[15] Hardie D.G. (2018). Keeping the home fires burning: amp-activated protein kinase. J. R. Soc. Interface.

[16] Woods A., Johnstone S.R., Dickerson K., Leiper F.C., Fryer L.G., Neumann D., Schlattner U., Wallimann T., Carlson M., Carling D. (2003). LKB1 is the upstream kinase in the AMP-activated protein kinase cascade. Curr. Biol.: CB.

[17] Hurley R.L., Anderson K.A., Franzone J.M., Kemp B.E., Means A.R., Witters L.A. (2005). The Ca2+/calmodulin-dependent protein kinase kinases are AMP-activated protein kinase kinases. J. Biol. Chem..

[18] Boudeau J., Baas A.F., Deak M., Morrice N.A., Kieloch A., Schutkowski M., Prescott A.R., Clevers H.C., Alessi D.R. (2003). MO25alpha/beta interact with STRADalpha/beta enhancing their ability to bind, activate and localize LKB1 in the cytoplasm. EMBO J..

[19] Tomizawa A., Hattori Y., Inoue T., Hattori S., Kasai K. (2011). Fenofibrate suppresses microvascular inflammation and apoptosis through adenosine monophosphate-activated protein kinase activation. Metab.: Clin. Exp..

[20] Kim J., Ahn J.H., Kim J.H., Yu Y.S., Kim H.S., Ha J., Shinn S.H., Oh Y.S. (2007). Fenofibrate regulates retinal endothelial cell survival through the AMPK signal transduction pathway. Exp. Eye Res..

[21] Feletou M., Vanhoutte P.M. (2006). Endothelial dysfunction: a multifaceted disorder (The Wiggers Award Lecture). Am. J. Physiol. Heart Circ. Physiol..

[22] Gluais P., Paysant J., Badier-Commander C., Verbeuren T., Vanhoutte P.M., Feletou M. (2006). In SHR aorta, calcium ionophore A-23187 releases prostacyclin and thromboxane A2 as endothelium-derived contracting factors. Am. J. Physiol. Heart Circ. Physiol..

[23] Smith W.L., DeWitt D.L., Garavito R.M. (2000). Cyclooxygenases: structural, cellular, and molecular biology. Annu. Rev. Biochem..

[24] Wong S.L., Leung F.P., Lau C.W., Au C.L., Yung L.M., Yao X., Chen Z.Y., Vanhoutte P.M., Gollasch M., Huang Y. (2009). Cyclooxygenase-2-derived prostaglandin F2alpha mediates endothelium-dependent contractions in the aortae of hamsters with increased impact during aging. Circ. Res..

[25] Shi Y., Feletou M., Ku D.D., Man R.Y., Vanhoutte P.M. (2007). The calcium ionophore A23187 induces endothelium-dependent contractions in femoral arteries from rats with streptozotocin-induced diabetes. Br. J. Pharmacol..

[26] Zhao L., Gao Y., Cao X., Gao D., Zhou S., Zhang S., Cai X., Han F., Wilcox C.S., Li L., Lai E.Y. (2017). High-salt diet induces outward remodelling of efferent arterioles in mice with reduced renal mass. Acta Physiol. (Oxf.).

[27] Huang Q., Wang Q., Zhang S., Jiang S., Zhao L., Yu L., Hultstrom M., Patzak A., Li L., Wilcox C.S., Lai E.Y. (2016). Increased hydrogen peroxide impairs angiotensin II contractions of afferent arterioles in mice after renal ischaemia-reperfusion injury. Acta Physiol. (Oxf.).

[28] Stehouwer C.D. (2004). Endothelial dysfunction in diabetic nephropathy: state of the art and potential significance for non-diabetic renal disease. Nephrol. Dial. Transplant..

[29] Chen Z.P., Mitchelhill K.I., Michell B.J., Stapleton D., Rodriguez-Crespo I., Witters L.A., Power D.A., Ortiz de Montellano P.R., Kemp B.E. (1999). AMP-activated protein kinase phosphorylation of endothelial NO synthase. FEBS Lett..

[30] Morrow V.A., Foufelle F., Connell J.M., Petrie J.R., Gould G.W., Salt I.P. (2003). Direct activation of AMP-activated protein kinase stimulates nitric-oxide synthesis in human aortic endothelial cells. J. Biol. Chem..

[31] Zhang W., Wang Q., Song P., Zou M.H. (2013). Liver kinase b1 is required for white adipose tissue growth and differentiation. Diabetes.

[32] Pieper G.M., Moore-Hilton G., Roza A.M. (1996). Evaluation of the mechanism of endothelial dysfunction in the genetically-diabetic BB rat. Life Sci..

[33] Bonekamp N.A., Volkl A., Fahimi H.D., Schrader M. (2009). Reactive oxygen species and peroxisomes: struggling for balance. BioFactors (Oxf., Engl.).

[34] Delille H.K., Bonekamp N.A., Schrader M. (2006). Peroxisomes and disease - an overview. Int. J. Biomed. Sci.: IJBS.

[35] Fransen M., Nordgren M., Wang B., Apanasets O. (2012). Role of peroxisomes in ROS/RNS-metabolism: implications for human disease. Biochim. Biophys. Acta.

[36] Ido Y., Carling D., Ruderman N. (2002). Hyperglycemia-induced apoptosis in human umbilical vein endothelial cells: inhibition by the AMP-activated protein kinase activation. Diabetes.

[37] Bertoluci M.C., Ce G.V., da Silva A.M., Wainstein M.V., Boff W., Punales M. (2015). Endothelial dysfunction as a predictor of cardiovascular disease in type 1 diabetes. World J. Diabetes.

[38] Pacher P., Beckman J.S., Liaudet L. (2007). Nitric oxide and peroxynitrite in health and disease. Physiol. Rev..

[39] Szabo C., Ischiropoulos H., Radi R. (2007). Peroxynitrite: biochemistry, pathophysiology and development of therapeutics. Nat. Rev. Drug Discov..

[40] Radovits T., Seres L., Gero D., Lin L.N., Beller C.J., Chen S.H., Zotkina J., Berger I., Groves J.T., Szabo C., Szabo G. (2007). The peroxynitrite decomposition catalyst FP15 improves ageing-associated cardiac and vascular dysfunction. Mech. Ageing Dev..

[41] Lopez-Lopez J.G., Moral-Sanz J., Frazziano G., Gomez-Villalobos M.J., Moreno L., Menendez C., Flores-Hernandez J., Lorente J.A., Cogolludo A., Perez-Vizcaino F. (2011). Type 1 diabetes-induced hyper-responsiveness to 5-hydroxytryptamine in rat pulmonary arteries via oxidative stress and induction of cyclooxygenase-2. J. Pharmacol. Exp. Ther..

[42] Bagi Z., Erdei N., Papp Z., Edes I., Koller A. (2006). Up-regulation of vascular cyclooxygenase-2 in diabetes mellitus. Pharmacol. Rep..

[43] Retailleau K., Belin de Chantemele E.J., Chanoine S., Guihot A.L., Vessieres E., Toutain B., Faure S., Bagi Z., Loufrani L., Henrion D. (2010). Reactive oxygen species and cyclooxygenase 2-derived thromboxane A2 reduce angiotensin II type 2 receptor vasorelaxation in diabetic rat resistance arteries. Hypertension.

[44] Kassan M., Choi S.K., Galan M., Bishop A., Umezawa K., Trebak M., Belmadani S., Matrougui K. (2013). Enhanced NF-kappaB activity impairs vascular function through PARP-1-, SP-1-, and COX-2-dependent mechanisms in type 2 diabetes. Diabetes.

[45] de Sotomayor M.A., Perez-Guerrero C., Herrrera M.D., Jimenez L., Marin R., Marhuenda E., Andriantsitohaina R. (2005). Improvement of age-related endothelial dysfunction by simvastatin: effect on NO and COX pathways. Br. J. Pharmacol..

[46] Yamada T., Fujino T., Yuhki K., Hara A., Karibe H., Takahata O., Okada Y., Xiao C.Y., Takayama K., Kuriyama S., Taniguchi T., Shiokoshi T., Ohsaki Y., Kikuchi K., Narumiya S., Ushikubi F. (2003). Thromboxane A2 regulates vascular tone via its inhibitory effect on the expression of inducible nitric oxide synthase. Circulation.

